# DNA G-Quadruplex Recognition In Vitro and in Live Cells by a Structure-Specific Nanobody

**DOI:** 10.1021/jacs.2c10656

**Published:** 2022-12-09

**Authors:** Silvia Galli, Larry Melidis, Sean M. Flynn, Dhaval Varshney, Angela Simeone, Jochen Spiegel, Sarah K. Madden, David Tannahill, Shankar Balasubramanian

**Affiliations:** † Cancer Research UK Cambridge Institute, Li Ka Shing Centre, Robinson Way, Cambridge CB2 0RE, U.K.; ‡ Yusuf Hamied Department of Chemistry, University of Cambridge, Cambridge CB2 1EW, U.K.; § School of Clinical Medicine, University of Cambridge, Cambridge CB2 0SP, U.K.

## Abstract

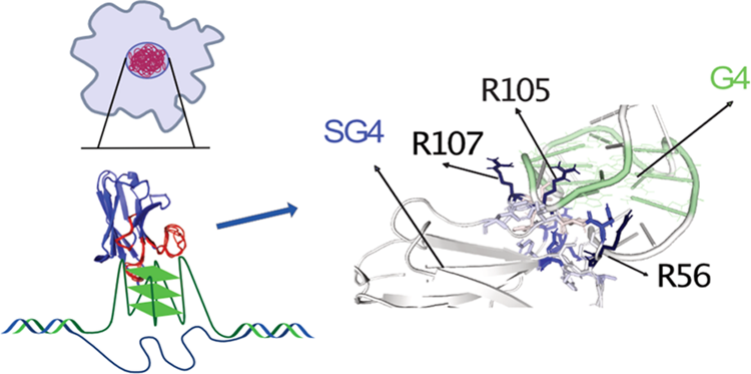

G-quadruplexes (G4s) are four-stranded DNA secondary structures that occur in the human genome and play key roles in transcription, replication, and genome stability. G4-specific molecular probes are of vital importance to elucidate the structure and function of G4s. The scFv antibody BG4 has been a widely used G4 probe but has various limitations, including relatively poor in vitro expression and the inability to be expressed intracellularly to interrogate G4s in live cells. To address these considerations, we describe herein the development of SG4, a camelid heavy-chain-only derived nanobody that was selected against the human Myc DNA G4 structure. SG4 exhibits low nanomolar affinity for a wide range of folded G4 structures in vitro. We employed AlphaFold combined with molecular dynamics simulations to construct a molecular model for the G4–nanobody interaction. The structural model accurately explains the role of key amino acids and *K*_d_ measurements of SG4 mutants, including arginine-to-alanine point mutations that dramatically diminish G4 binding affinity. Importantly, predicted amino acid–G4 interactions were subsequently confirmed experimentally by biophysical measurements. We demonstrate that the nanobody can be expressed intracellularly and used to image endogenous G4 structures in live cells. We also use the SG4 protein to positionally map G4s in situ and also on fixed chromatin. SG4 is a valuable, new tool for G4 detection and mapping in cells.

## Introduction

**Figure 1:**
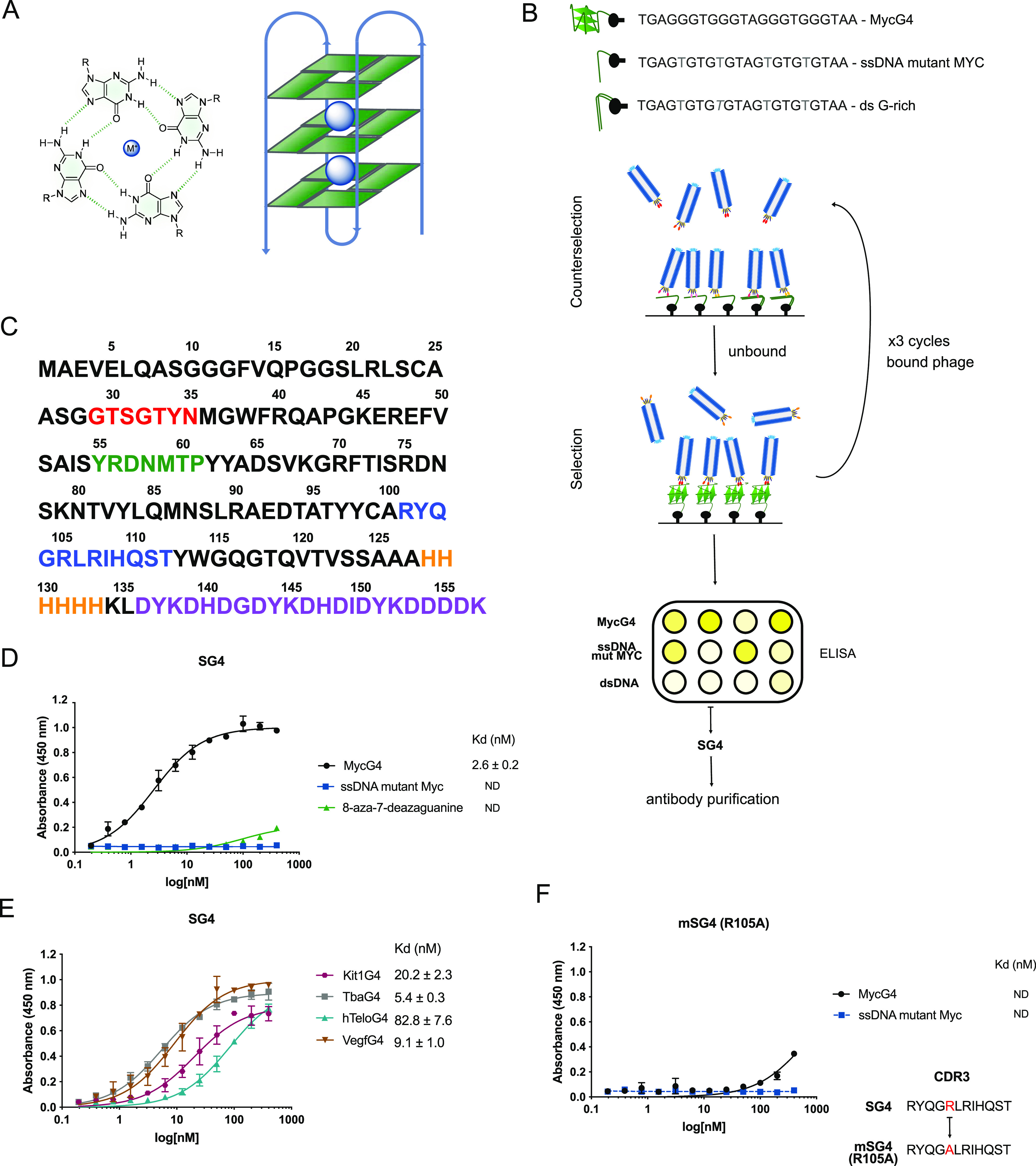
(A) Schematic representation of a G-tetrad (four guanines in green), stabilized by a monovalent cation (M^+^), and of a G-quadruplex. (B) Workflow of phage display screening of a nanobody library and validation through nonadsorbent phage ELISA, leading to the selection of G4-binding SG4. Sequences of the oligonucleotides used in the screening are reported. A counterselection step against single-stranded and double-stranded DNA negative controls was followed by three rounds of incubation with MycG4. (C) The amino acid sequence of the SG4 nanobody. Complementarity-determining regions (CDRs) highlighted in red (CDR1), green (CDR2), and blue (CDR3). The nanobody is tagged with 6xHIS (orange) and with a 3xFLAG tag (purple). The amino acid position is reported above the sequence. (D) SG4 binding curves to MycG4 and negative controls ssDNA mutant Myc and 8-aza-7-deazaguanine corresponding oligonucleotide, determined by ELISA. (E) ELISA SG4 binding curves to different G4 topologies: Kit1G4 and VegfG4 (parallel), TbaG4 (antiparallel), and hTeloG4 (hybrid). (F) ELISA mSG4 R105A binding curves to MycG4 and negative control ssDNA mutant Myc. The sequence of CDR3 carrying the mutation from arginine to alanine is reported. Dissociation constants (*K*_d_) are indicated in nanomolar; in some cases, they could not be determined (ND). Error bars represent the standard error of the mean (s.e.m.) calculated from two replicates.

DNA comprising particular G-rich sequences can fold into four-stranded G-quadruplex (G4) secondary structures, which are formed from stacking of planar guanine-tetrads each assembled from Hoogsteen hydrogen bonding and stabilized by a central monovalent cation coordinated to the O6 of guanines (Figure 1A).^1,2^ A single strand of DNA can fold into an intramolecular G4, which is thermally very stable at physiological salt, pH, and temperature.^3^ Several hundreds of thousands of G4s have potential to be formed based on sequencing experiments on human genomic DNA.^4^ Data from biological experiments have revealed that DNA G4s have important roles linked to gene regulation and telomere biology.^2^ It has also been demonstrated that DNA G4s have potential to be recognized in cells by proteins that include transcription factors and chromatin remodelers.^5−7^ To further our understanding of G4s and their biological role, it has been of vital importance to develop molecular probes that recognize DNA G4 structures with high affinity and structural specificity. We previously developed the engineered single-chain antibody (scFv) BG4,^8^ which recognizes G4s and has been applied to image^8−12^ and also sequence^13−16^ G4s in human cells and chromatin after fixing cells. While such approaches provided helpful insights, it was not possible to express the probe BG4 in live cells, as scFvs commonly do not fold properly in the reducing environment of mammalian cells.^17^ Furthermore, BG4 is also difficult to produce in quantities that would enable structural studies.

We considered engineering a probe derived from the next-generation antibody class from camelids, called nanobodies, to overcome key limitations of BG4 and interrogate G4 formation in cells. Nanobodies are relatively small antibodies (∼12 to 15 kDa), with antigen recognition mediated by three protein domains called complementarity-determining regions (CDRs).^18,19^ CDR3, the longest, is thought to play a central role in antibody–antigen interactions to enable the recognition of epitopes inaccessible by conventional antibodies.^20,21^ Compared to BG4, nanobodies are smaller in size, express easily in bacteria, and can be easily engineered to change their chemical and molecular features due to their simpler structure.

Herein, we describe the generation and characterization of SG4, a novel nanobody with high affinity and specificity for a range of G4s. We employed Alphafold in combination with molecular dynamics to create structural models of G4 recognition by the nanobody SG4, which were validated experimentally by single-residue mutations. Importantly, we were able to deploy SG4 in situ by expression in human cells and demonstrate its use as a molecular probe to detect and map G4s in the chromatin of human cancer cell lines.

## Results and Discussion

### Isolation and In Vitro Binding Characteristics of a G4 Nanobody

To isolate nanobodies that selectively recognize G4 structures, a DNA oligonucleotide for a parallel G4 from the human *MYC* upstream promoter,^22^ designated here as MycG4, was used as bait to screen the Hybrigenics Services SAS hsd2Ab library of synthetic human nanobodies by phage display^23^ (Figure 1B). The MycG4 has been well characterized for stability and folding kinetics, and its structure has been resolved at atomic resolution by NMR spectroscopy and X-ray crystallography.^22,24,25^ Three rounds of positive selection against a folded, biotinylated MycG4 oligonucleotide were performed. Nonspecific binders were removed prior to each round by negative selection against single-stranded MycG4 with G-to-T substitutions, which disrupt G4 formation, and G-rich dsDNA biotinylated oligonucleotides. Nonspecific binders were further deselected using yeast tRNA, salmon sperm DNA, and random primer single-stranded DNA as competitors in rounds 2 and 3. From the initial library of 3 × 10^9^ clones, 54 independent positive nanobodies were recovered as determined by sequencing. The clone, SG4, with the highest ratio of binding affinity (MycG4 vs negative controls) in phage ELISA assays, in which the target is incubated with the nanobody displayed on phage surfaces, was selected for further characterization. The amino acid sequence of SG4 is reported in Figure 1C.

SG4 was next engineered to carry the 3xFLAG epitope tag, and the FLAG-tagged SG4 protein was then expressed and purified from *Escherichia coli* using immobilized metal affinity chromatography and elution with imidazole (see Materials and Methods in Supporting Information), resulting in a good yield of ∼0.7 mg from 100 mL of starting culture. The size of purified SG4 protein was ∼18 kDa by SDS-PAGE and is in good agreement with the predicted molecular weight of 17.7 kDa (157 amino acids, Figure S1). SG4 binding affinities for G4s were then measured by ELISA using DNA oligonucleotides with human genomic sequences, which have previously been shown to fold into different G4 structural topologies that included MycG4, Kit1G4, VegfG4 (parallel propeller), TbaG4 (antiparallel), and hTeloG4 (mixed parallel/antiparallel propeller), along with a comparison with control oligonucleotides unable to fold into a G4 in vitro (Figure 1D,E).^8,22,26−28^ G4 structure formation from the oligonucleotides was confirmed by circular dichroism (CD) spectroscopy (Figure S2A,B). SG4 binds to MycG4 with low nanomolar affinity (apparent *K*_d_ = 2.6 nM), and poor binding was observed with mutated MycG4 or MycG4 in which the central Gs were substituted with 8-aza-7-deazaguanine to disrupt G4 formation (Figure 1E). SG4 also has a low nanomolar affinity for several different G4 topologies, ranging from 5.4 nM for TbaG4 (antiparallel), 9.1–20.2 nM for VegfG4 and Kit1G4 (parallel), and 82.8 nM for hTeloG4 (Figure 1E). SG4, like BG4,^8^ therefore recognizes a range of G4 structural types.

CDR3 is the most important CDR for binding in nanobodies.^20,21^ The CDR3 of SG4 predominantly contains positive or polar amino acids (RYQGRLRIHQST). Basic arginines and lysines have been shown to be key amino acids in many protein–nucleic acid interactions. They are enriched in DNA binding proteins together with histidine residues, which are neutral at a physiological pH of 7.4.^29−31^ Therefore, we next tested the effects on SG4 binding affinity by mutating the single arginines in CDR3 to alanines (R101A, R105A, R107A). FLAG-tagged mutants of SG4 were expressed and purified from *E. coli* as for SG4, and when tested by ELISA, R105A and R107A SG4 mutants showed a substantial loss of affinity (*K*_d_ > 200 nM) for MycG4, whereas R101A showed little reduction (1.5 fold) in its binding affinity (Figures 1F and S3). Instead, when histidine in CDR3 was mutated to alanine (H109A), no change in binding affinity was detected (Figure S3). Collectively, these mutagenesis experiments identified two critical, positively charged arginines required for G4 recognition by the SG4 nanobody.

### Modeling the Interaction of SG4 and MycG4

**Figure 2:**
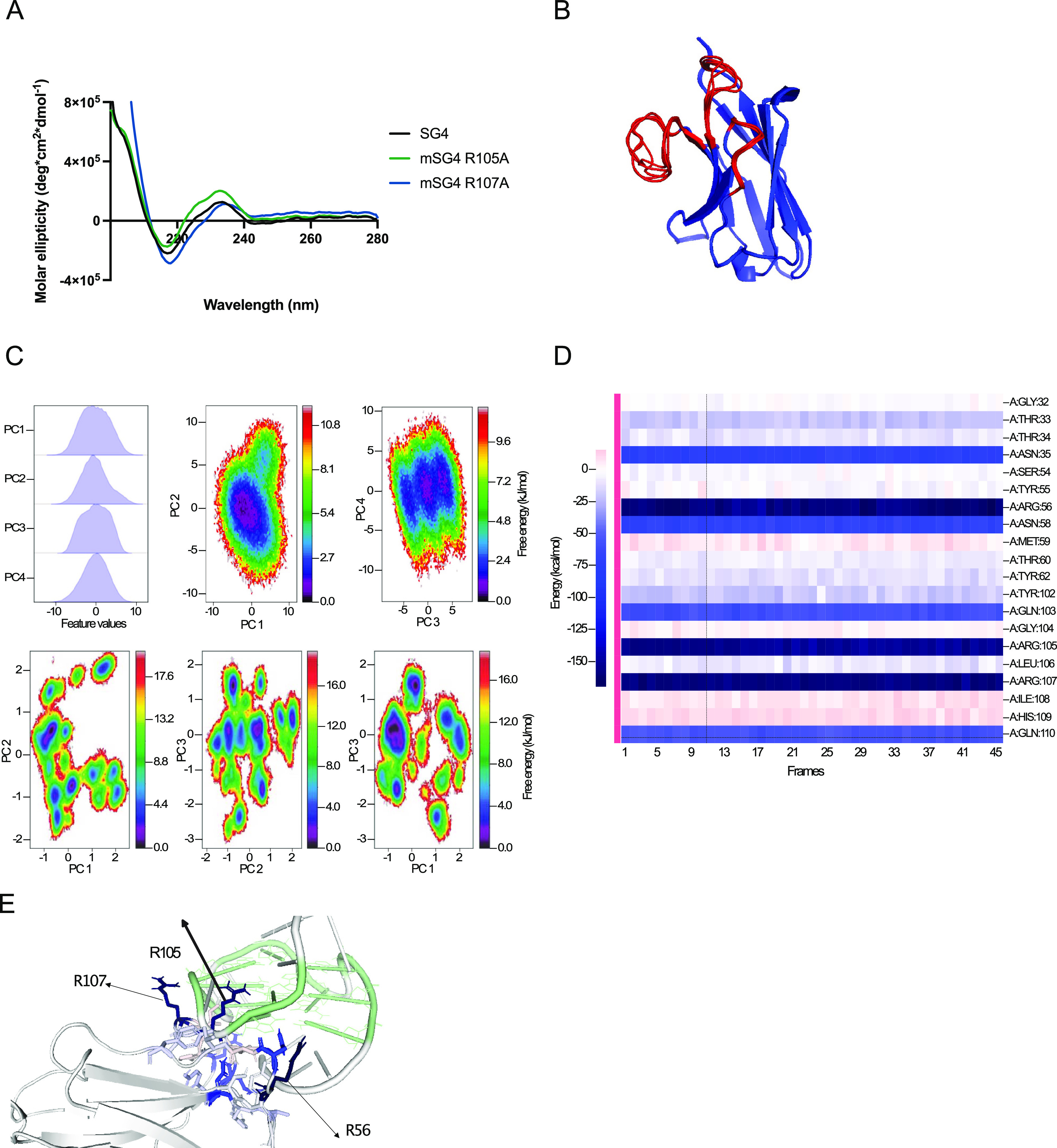
(A) SG4, mSG4 R105A, and mSG4 R107A secondary structures determined by CD spectroscopy (200–280 nm) showing a characteristic β-sheet with a negative peak at 218 nm and a positive peak at 200 nm. Units are measured in molar ellipticity. (B) Overlap of Alphafold 2.0 top 5 structure predictions, CDRs in red and scaffold in blue. (C) First four principal components and projections between 1–2 and 3–4, and time-lagged cross-correlation of the combined 5 μs simulation of SG4 with a time lag of 5 ns. (D) MMGBPA analysis of the binding mode of SG4 with MycG4 throughout MD simulations of 500 ns (in 50 frames) with the individual contribution of residues. (E) 3D frame with residues colored according to energy contribution, G4 in light green.

We employed molecular modeling to gain structural insights into the recognition of MycG4 by the SG4 nanobody. As there is no experimentally derived structure for SG4, we used Alphafold2^32,33^ to generate 3D protein structure predictions for wild-type SG4 and two mSG4 variants (R105A and R107A) that exhibit compromised G4 binding affinity. This resulted in five conformations for each nanobody. Consistent with existing nanobody X-ray crystal structural data and NMR,^20,34^ the main scaffold for all three nanobodies was structurally similar to the established β-sheet conformation, also supported by the circular dichroism spectra of the proteins (Figure 2A). Due to the large variability in CDR amino acid sequences across antibodies, these regions are expected to pose a challenge for Alphafold2 and other machine learning techniques, so we used molecular dynamics simulations (MDS) to sample the conformational space of the SG4 CDRs (Figures 2B and S4A,B). Each of the five Alphafold2 output conformations was used to initiate a 1 μs long MDS (Figure S4C), combining all independent simulations into one data set. Using principal component analysis of the first two components does not suggest any ordered stable structure in the CDRs (Figure 2C). Examining the landscape in a time-dependent manner^35^ suggests possible metastable energy states in the conformation space of the CDR regions in this short timescale; however, longer simulations are required to confidently identify the overall dynamics of CDRs. Most stable confirmations identified during these simulations were then used for conformations in computational docking experiments.

Given the conformational flexibility of both SG4 (shown by MDS) and MycG4 (shown by NMR pdb; 1XAV), potential interaction modes between them were explored by computational docking between multiple conformations of both SG4 and MycG4. For SG4, these included the five conformations of the Alphafold output and five extracted from MDS (centers of tICA landscape clusters). For MycG4, the first 10 structures derived from NMR spectroscopy (1XAV pdb) were used to widen the conformational landscape of potential docking interactions. All combinations of SG4 with MycG4 were computationally docked using High Ambiguity Driven protein–protein DOCKing (HADDOCK 2.4).^36,37^ Each combination resulted in multiple clusters of suggested docking, and only those with docking scores lower than −120 (arbitrary units) were selected for further analysis. This data set, which formed two clusters of similar binding scores, was subject to further MDS to evaluate the suggested binding modes and the per residue energy decomposition calculated using the gmx_MMPBSA pipeline.^38^ This modeling approach identified three key amino acids contributing the highest energy involved in binding (R56, R105, R107; Figure 2E, dark blue). Two of them that were already identified independently by single amino acid substitution experiments, R105 and R107 in CDR3, had the highest contribution in MDS, and their mutation (R105A and R107A) showed significantly decreased affinity by ELISA, both without detectable *K*_d_ values (>200 nM), and R105A was the more damaging of the two (Figures 2D,E, S3, and S4D).

Additionally, the third amino acid highlighted in our modeling was R56 in CDR2, by interacting with the antidiametrical groove to that of R105 (Figure 2D). We evaluate the predicted R56–G4 interaction experimentally by engineering an SG4 variant carrying an R56A mutation. After confirming a similar secondary structure with SG4 and the other mutants through CD spectroscopy (Figure S5A), we found that the R56A mutation significantly reduced the binding affinity for MycG4 by ELISA (estimated *K*_d_ > 200 nM; Figure S5B). Further exploration of the modeling data suggests that amino acids R101 and H109 in CDR3 should have a low contribution to binding (<−10 kcal/mol), which was validated by our experimental data for the mutant SG4s, which showed that these amino acids had only small effects on SG4 binding affinity by ELISA (R101A *K*_d_ 4.0 ± 0.5 nM, H109A *K*_d_ 2.9 ± 0.2 nM; Figure S4E). Together, our experimental and computational analyses show that both CDR3 and CDR2 residues contribute to SG4–MycG4 binding. The computational analysis of SG4 bound to MycG4 has provided a prediction of key protein–DNA interactions that showed remarkable agreement with experimental data along with important insights into G4 recognition. It also provides a method to probe structural information on systems difficult to study experimentally.

### Detecting G4s in Human Chromatin

We next explored whether the purified SG4 nanobody could capture G4 structures from human cells. For this, we used SG4 to capture G4s, followed by sequencing of the DNA fragments containing the captured G4s, using fixed chromatin (G4 ChIP-seq) samples isolated from two human cancer cell lines (K562 and U2OS). First, we verified that a selection of regions known to fold into G4s in chromatin could be captured and identified using SG4 by ChIP-PCR experiments. This showed that SG4, but not mSG4 R105A, enriches known G4-folded regions from chromatin^39^ and confirms that G4 recognition by SG4 is specific and not through nonspecific interactions with the nanobody scaffold (Figures S6 and S7). Next, G4 ChIP-seq was used to capture genome-wide G4s on chromatin from K562 and U2OS cancer cell lines, resulting in 5531 and 10621 high-confidence regions, respectively, that were previously shown in vitro to have G4-folding potential^4^ (Figure S8A,C). There was also significant enrichment (∼20%) in open-chromatin regions and gene-regulatory regions (Figures S8B,D and S9A,B). A range of G4 structural variants, such as 3 G-tetrads with a loop of 1–7 bases (G3L1-7)_4_, longer loops, simple bulges, or 2 G-tetrad forms,^40^ examined were found to be significantly enriched by SG4 when compared to what would be expected at random (Figures S9C and S10). Overall, these results show that SG4 can successfully map a range of G4s in the genome within cellular chromatin.

**Figure 3:**
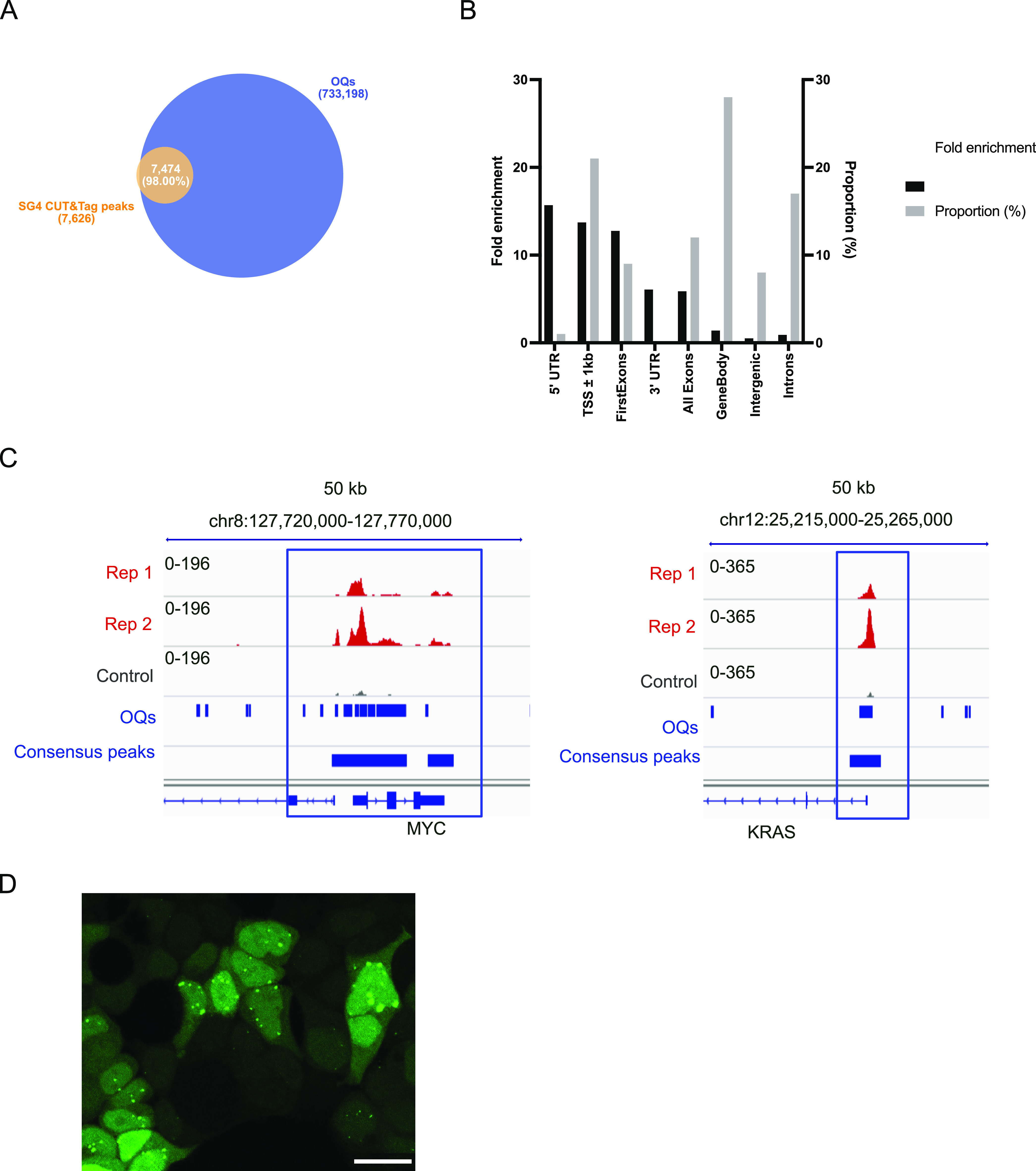
(A) Venn diagrams of the overlap of SG4 regions identified through CUT&Tag (SG4 CUT&Tag peaks) with sequences previously identified as capable of folding into a G4 structure in vitro (so-called observed G4 sequences and referred to as OQs) (4) in HEK293T. (B) Fold enrichment over random (black bars) and proportion (gray) of SG4 CUT&Tag consensus regions across different genomic features in HEK293T. (C) Genome browser screenshots for biological replicates of CUT&Tag for SG4-GFP-FLAG-expressing HEK293T cells (Rep1 and Rep2), and control HEK293T (cells not expressing SG4-GFP-FLAG) using an anti-FLAG primary antibody. Strong peaks are shown for the *MYC* and *KRAS* promoter G4 regions in cells expressing SG4-GFP-FLAG upon doxycycline treatment. (D) Confocal live cell imaging of SG4 foci in the nuclei of HEK293T cells expressing SG4-GFP. The scale bar is 20 μm.

A particular advantage of nanobodies is their ability to bind endogenous molecular targets in living cells. We therefore deployed SG4 to capture G4s from live cells. For this, we engineered an SG4-GFP-FLAG fusion protein construct carrying a nuclear localization signal under the control of a TET-on gene expression system. We stably transfected human embryonic kidney 293T cells and induced fusion protein expression by addition of doxycycline to the media for 72 h. To confirm that SG4 was bound to known G4 sites when expressed in living cells, we adapted a method that exploited a Tn5 transposase fused to protein A to bind endogenous antibody-bound sites and insert DNA sequencing adapters (G4 CUT&Tag).^14,16^ We thus used an anti-FLAG secondary antibody and an anti-rabbit tertiary antibody to recruit protein A-Tn5 transposase to the SG4-bound site and prepared sequencing libraries from fragments tagmented by Tn5 transposase. Endogenously expressed SG4 bound to 7626 high-confidence regions in 2/2 biological replicates and 3/3 technical replicates. In total, 98% of these (7474/7626) overlapped with sites with potential of folding into G4s in vitro (Figure 3A).^4^ SG4-bound regions were enriched in the 5′ UTR and near the TSS of genes (Figure 3B).

Endogenously expressed SG4 recognized known G4s in the upstream regulatory regions (promoters) of the *MYC* and *KRAS* oncogenes (Figure 3C). We next imaged live cells expressing the SG4-GFP-FLAG protein by fluorescence confocal microscopy for GFP and observed distinct nuclear foci, which are characteristic of G4s detected by BG4 (Figure 3D).^8,41^ Taken together, these experiments demonstrate that SG4 can detect and probe G4s within living cells.

## Conclusions

Here, we describe the generation and characterization of SG4, a novel nanobody molecular probe, for the detection of naturally occurring DNA G4 structures in human chromatin. Using a combination of de novo protein structure prediction and molecular dynamics along with single-residue mutation, we present for the first time molecular insights into an antibody–G4 binding interaction and identify critical amino acids required for low nanomolar affinity. The nanobody SG4 can be deployed in parallel with BG4 to cross-validate findings based on G4 structure formation in the human and other cell types. The availability of SG4, a high affinity and specific nanobody, in addition to validating and extending G4 landscapes seen with BG4, overcomes the limitations of scFvs in cellular expression experiments.

## Supporting Information Available

Detailed description of experimental procedures; materials; and additional figures as mentioned in the text (PDF)

## Abbreviations

G4: G-quadruplex
scFv: single-chain variable fragment
*K*_d_: dissociation constant
CDR: complementarity-determining region
ELISA: enzyme-linked immunosorbent assay
SDS–PAGE: sodium dodecyl sulfate–polyacrylamide gel electrophoresis
CD: circular dichroism
nM: nanomolar
MDS: molecular dynamics simulation
HADDOCK: High Ambiguity Driven protein–protein DOCKing
ChIP-seq: chromatin immunoprecipitation sequencing
GFP: green fluorescent protein
TET-on: tetracycline-on
Tn5: transposon 5
CUT&Tag: cleavage under targets and tagmentation
UTR: untranslated region
TSS: transcription start site
OQ: observed G-quadruplex sequence
MMPBSA: molecular mechanics Poisson–Boltzmann surface area
MMBGSA: molecular mechanics surface area
NMR: nuclear magnetic resonance
