# Supplementary material for: DNA G-Quadruplex Recognition In Vitro and in Live Cells by a Structure-Specific Nanobody
Source: J Am Chem Soc. 2022 Dec 9;144(50):23096–103. doi: 10.1021/jacs.2c10656 (PMC9782783; doi:10.1021/jacs.2c10656)
Supplement: Supplementary file 1 — ja2c10656_si_001.pdf [file ja2c10656_si_001.pdf]

DNA G-QUADRUPLEX RECOGNITION IN VITRO AND IN LIVE CELLS BY A STRUCTURE-SPECIFIC NANOBODY

Silvia Galli[1], Larry Melidis[1,2], Sean M. Flynn[1], Dhaval Varshney[1], Angela Simeone[1], Jochen Spiegel[1,2], Sarah K. Madden[2], David Tannahill[1], Shankar Balasubramanian[1,2,3*]

1Cancer Research UK Cambridge Institute, Li Ka Shing Centre, Robinson Way, Cambridge, CB2 0RE, UK.
2Yusuf Hamied Department of Chemistry, University of Cambridge, Cambridge, CB2 1EW, UK
3School of Clinical Medicine, University of Cambridge, Cambridge, CB2 0SP, UK
*Correspondence: sb10031@cam.ac.uk (S.B.)

SUPPORTING INFORMATION

Materials and Methods

Phage display antibody selection

A naive synthetic library NaLi-H1 (Nanobody Library-Humanized 1) was screened against G4 structure MycG4 for the *in vitro* selection of the G4-specific nanobody by Hybrigenics Services SAS (Hybrigenics Services SAS, 3-5 Impasse Reille, 75014 Paris, France, www.hybrigenics-services.com). The biotinylated oligonucleotides have been folded in 10 mM Tris pH 7.4, 100 mM KCl, and 0.1% Tween-20, annealed by heating to 95 °C for 10 minutes followed by slow cooling down to 23 °C (sequences reported in the table below). The screening was performed in three rounds of phage display against MycG4 (50 nM in the first round, 10 nM in second and third rounds). Before each round of selection, nanobodies were counterselected by incubating phages displaying the nanobodies with 50 nM dsDNA and ssDNA bound to streptavidin magnetic beads to select the library against non-specific binders. During the second and third rounds the nanobodies were presented to 10 nM MycG4 in presence of 5 nM and 10 nM of a mix of competitors (yeast tRNA, salmon sperm DNA, random primer single-stranded DNA). The nanobody is cloned in pHEN2 phagemid carrying also the sequences for a 6xHIS and a triple MYC tag. We changed the tag of the novel G4-binding nanobody to 3xFLAG to have a probe comparable to scFV BG4, also FLAG-tagged. The plasmid was digested with *NotI* and *BglII* (NEB) and 3xFLAG tag was inserted by Gibson Assembly according to the manufacturer protocol (Gibson Assembly Master Mix, E2611S, New England Biolabs). The dead mutants mSG4 was generated by mutation of a single amino acid to alanine through side-directed mutagenesis (Q5 site-directed mutagenesis kit, E0554S, New England Biolabs).

Nanobodies expression and purification in E. coli

BL21 (DE3) *E. coli* chemically competent cells were transformed with pHEN2 plasmid carrying either SG4 wild type or the mutated sequences. Single colonies were inoculated in Terrific Broth medium the addition of 1% glucose and 100 μg/ml ampicillin. After overnight incubation, the starting culture were transferred into Terrific Broth medium containing 0.1% glucose and 100 μg/ml ampicillin, incubated at 37 °C until bacterial OD600 reached a value in the range of 0.6-0.8. After that, bacteria were induced with 0.5 mM IPTG for ~16 h at 28 °C. Bacterial lysed fraction containing the nanobody was loaded on Proteus 1-step batch Midi Spin columns (Generon) with Nickel resin (Sigma), and incubated at 4 °C for 1 hour, then the nanobodies were eluted with 250 mM imidazole, and dialyzed against PBS at 4 °C using GeBaflex tubes (Generon). The expression and purification of the recombinant proteins from E. coli was checked through SDS-PAGE followed by Coomassie staining (Thermo Fisher Scientific NuPAGE 4-12% Bis-Tris gel, run in Invitrogen NuPAGE MOPS SDS Running Buffer (20x). Staining in Instantblue Coomassie Protein Stain ab11921.

CD spectroscopy

CD spectroscopy was performed using Chirascan CD spectropolarimeter (Applied Photophysics). Scans of the DNA oligonucleotides reported in Table 1 were performed in triplicates at a concentration of 10 μM (10 mM Tris HCl, 100 mM KCl, pH 7.4), in the range of wavelengths from 220 to 340 nm in a 1 mm cuvette, with readings taken every 1 nm with 1 second per point. In CD melting experiments, DNA oligonucleotides were analyzed at the concentration of 10 μM (10 mM KCl, 10 mM lithium cacodylate, pH 7.4), in three replicates. The temperature was increased from 20 to 92 °C in smooth ramp mode at ramp-rate of 1 °C per min with 1 second per point. The nanobody SG4 WT and mutants mSG4s (R105A, R107A, R56A) were scanned in triplicates at 10 μM concentration (PBS pH 7.4) at 30°C in a 1 mm cuvette between 200-280 nm, with readings taken every 0.5 nm with 1 second per point. Data were smoothed using Chirascan software, and plotted with GraphPad Prism 9.

Enzyme-linked immunosorbent assay (ELISA)

Streptavidin-coated 96-well plates (Thermo Fisher) were incubated with biotinylated oligonucleotides diluted in 100 mM KCl, 50 mM H_2KPO_4. After blocking with 3% BSA, plates were incubated for 1 hour with serial dilutions of protein (from 400 to 0 nM diluted in 3% BSA), followed by 1 hour incubation with anti-FLAG tag HRP-conjugated antibody (ab1238, Abcam). The HRP substrate TMB (3,3′,5,5′- tetramethylbenzidine, Roche) was added to each well to allow detection. The intensity of the signal was measured as absorbance at 450 nm on a PHERAstar microplate reader (BMG Labtech). Data was fitted to a one-site specific binding model and binding affinity was assessed as equilibrium dissociation constant (Kd), calculated using GraphPad Prism (GraphPad Software Inc.).

G4 ChIP-seq library preparation

G4 ChIP-Seq was performed on K562 and U2OS fixed prepared as previously reported in Shen J., *et al* (1): three technical replicates were performed for each biological replicate per cell line. The amount of BG4 employed is ~200 ng as described previously (2), while ~ 750 ng of SG4 and mSG4 were used. The amount of DNA precipitated was measured with Qubit 3.0, while enrichment of G4s structures was assessed by qPCR. Libraries for single-end sequencing were prepared with Nextera XT primer adaptor.

SG4 eukaryotic expression

For cellular expression, a SG4 nanobody fusion with GFP carrying a SV40 nuclear localization sequence (NLS) and FLAG-tagged (see map) was synthesized as a gBlock Gene Fragment (Integrated DNA Technologies) and inserted in to the PiggyBac transposon in the pCLIIPI-BP eukaryotic expression vector (a gift from M. Narita, CRUK CI, UK) (3) using NEBuilder HiFi DNA assembly (NEB E5520S). pCLIIPI-BP carries a doxycycline-inducible promoter and a puromycin cassette. Plasmid DNA was prepared using a Plasmid Plus Midi Kit (Qiagen 12943). Plasmid DNA and PiggyBac transposase (mPB) were transfected into HEK293T cells using TransIT-293 (Mirus Bio MIR 2700), using a 5:1 ratio of transposon to transposase, and stably transfected cells were selected in 1 μg/ml puromycin for 14 days. Cells were maintained in DMEM supplemented with 10% tetracycline-free FBS (biosera FB-1001T) and expression of the SG4-GFP fusion was achieved by addition of 1 μg/ml doxycycline for 72 hours. Live cells were then imaged for GFP on a Leica SP5 confocal microscope at 40x magnification. Binding of the SG4-GFP fusion protein to the MYC or KRAS promoter was assayed by CUT&Tag, with three technical replicates (different cells from the same flask) for each of two biological replicates (different passages). Bulk CUT&Tag was performed as previously described (14), with the exception that anti-FLAG (Cell Signaling 2368; 1:100) was used as a primary antibody. Peaks were visualized on the IGV genome browser.

SG4-GFP fusion protein map

MAEVELQASGGGFVQPGGSLRLSCAASGGTSGTYNMGWFRQAPGKEREFVSAISYRDNMTPYYADSVKGRFTISRDNSKN
TVYLQMNSLRAEDTATYYCARYQGRLRIHQSTYWGQGTQVTVSSSRADPKKKRKVSGGGSGGSGSMDAKSLTAWSMVSK
GEELFTGVVPILVELDGDVNGHKFSVSGEGEGDATYGKLTLKFICTTGKLPVPWPTLVTTLTYGVQCFSRYPDHMKQHDF
FKSAMPEGYVQERTIFFKDDGNYKTRAEVKFEGDTLVNRIELKGIDFKEDGNILGHKLEYNYNSHNVYIMADKQKNGIKVN
FKIRHNIEDGSVQLADHYQQNTPIGDGPVLLPDNHYLSTQSALSKDPNEKRDHMVLLEFVTAAGITLGMDELYKGGSGDY
KDHDGDYKDHDIDYKDDDDK

SG4 nanobody - SV40 nuclear localization sequence - Linker - GFP - FLAG epitope tag

Human reference genome and relative genomic annotations

Human genome reference hg38 fasta file was downloaded from UCSC database (hgdownload.cse.ucsc.edu/goldenPath/hg38/bigZips/hg38.fa.gz). Genomic annotations (gtf file) were downloaded from Genecode project portal (ftp.ebi.ac.uk/pub/databases/gencode/Gencode_human/release_28/gencode.v28.annotation.gtf.gz, Release 28 GRCh38.p12). Genomic regions used to assess enrichment of G4 sites (i.e. exons, introns, intergenic regions, 3'UTR, 5'UTR and 58381 promoters of all coding and not coding genes defined as TSS ± 1000bp) were extracted from the gtf file.

Sequencing Data processing

Sequenced libraries were Quality Checked (FastQC) and trimmed from Illumina sequencing adaptors by cutadapt (cutadapt -q 20 -O 3 -a CTGTCTCTTATACACATCT). Resulting reads were aligned to the human genome (hg38) using BWA-MEM. The alignments bam files were de-duplicated (picard MarkDuplicates) and used for generating genome tracks of G4 signal normalized to total library size (reads per million, RPM) for visual inspection. Local enrichments were identified calling peaks with MACS2 (options: -q 0.01 --keep-dup all) on the de-duplicated bam and the relative input library. G4 consensus regions of each biological replicate were obtained as the regions observed in 2 out of the 3 technical replicates (multiIntersectBed; using bedtools). The final cell-type consensus was defined as the regions reproducibly observed in 2 of the 3 biological replicates. The cell-type consensus was then compared to the OQs (4) and to accessible sites (1). CUT&Tag libraries were similarly processed as previously described (5) peaks were called using seacr 1.3 identifying the top 1% of regions by AUC, applying stringent threshold (6).

Characterization of G4 fold-enrichments at sites of interests

G4 fold-enrichments over random chance were evaluated at various sites of interest by using the Genomic Association Tester (GAT, https://gat.readthedocs.io/en/latest/contents.html, 1000 randomisations) restricting the analysis the human whitelist.

Structure prediction

The predicted structures of the nanobodies were generated using Colabfold, Alphafold1 using MMseqs2 (7), using Amber to relax the primary output structures, the 5 relaxed structures produced undergo 1 μs long molecular dynamics simulations.

Molecular dynamics

Molecular dynamics for both the nanobodies as well as the complexes were performed using Gromacs2021 (8) the amber14sb bcs1 DNA forcefield. Solute was placed in cubic box with periodic boundary conditions at least 1 nm away from the boundary, TIP3P Water was added along with and NaCl to simulate concentration of 50 nM. Initial minimisation was carried to at least 1000 kJ mol^{-1} nm^{-1} or 50 000 steps followed by heating and NVT equilibration for 1000 ps using V-rescale modified Berendsen thermostat, at 310K. All

simulations use 2 fs time step and Parrinello–Rahman pressure coupling and PME electrostatics at 1.0 nm cut-off. PCA and TiCA plots were produced with Pyemma 2.5.7 (9)

Docking

Docking was performed using High Ambiguity Driven protein-protein DOCKing (HADDOCK 2.4) (10) on the WeNMR-EOSC Ecosystem (11), between the 10 first NMR structures of MycG4 and 10 structures for each nanobody, suggesting the whole length of the DNA as active residues and the CDRs for the nanobodies. All other parameters are set to default for nucleic acid-protein interaction. Resulted proposed docking structures from HADDOCK, undertake a further 500 ns MD with the same parameters as before (KCl instead of NaCl). Gmx_MMPBSA(Molecular mechanics Poisson-Botlzmann surface area) (12) was used to analyze the resulted trajectories and the contribution of each residue during the above simulations (MM/GBSA calculations).

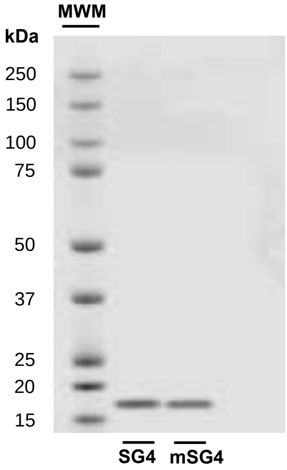

Figure S1. SDS-PAGE of purified nanobodies SG4, mutant mSG4 R105A (lane 2 and 3), and molecular weight marker (lane 1), stained with Instablue Coomassie staining. The nanobodies ran at around 18 kDa as predicted. Lefthand side molecular weights of the protein ladder in lane 1 (KDa).

S2B

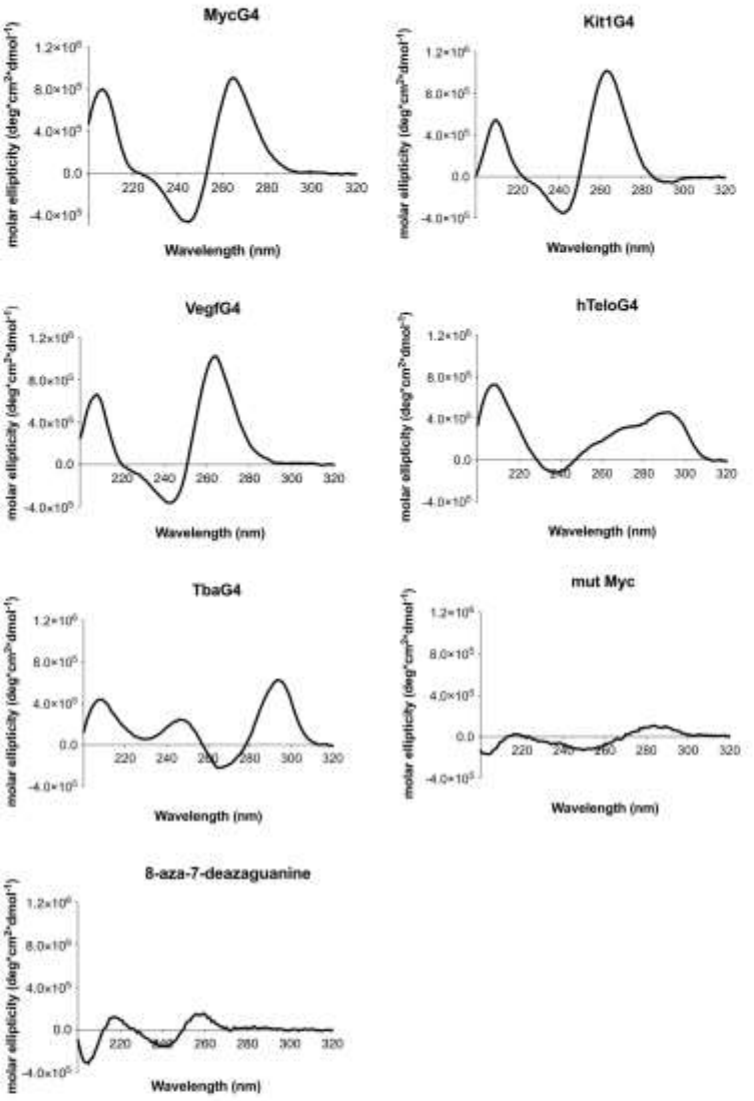

S2B

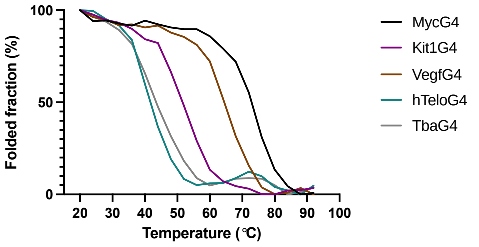

Figure S2. (A) Structures of the oligonucleotides employed in ELISAs analyzed in the presence of 100 mM KCl, at a concentration of 10 μM in Tris-HCl buffer using CD spectroscopy (220-340 nm). The spectra of MycG4, Kit1G4, and VegfG4 are typical of a parallel G-quadruplex with a positive peak at ~ 260 nm and a negative peak at ~ 240 nm. The spectrum of TbaG4 indicate the formation of an antiparallel G-quadruplexes with positive peaks at ~ 250 nm and ~ 290 nm and a negative peak at ~260 nm. The spectrum of hTeloG4 is typical of a hybrid parallel/antiparallel G-quadruplex with a positive peak at ~ 290 nm and a negative peak at ~ 240 nm. The spectra of ssDNA and 8-aza-7-deazaguanine used as negative control show a positive peak at ~280 nm and a negative peak at ~ 250 nm, not corresponding to any G-quadruplex topology. Units are measured in molar ellipticity. (B) Normalized CD melting temperature curves of oligonucleotides folded into G4s at the concentration of 10 μM in 10 mM KCl 10 mM lithium cacodylate. MycG4, Kit1G4, and VegfG4 (parallel G4s) were analyzed at 264 nm, while hTeloG4 and TbaG4 at 295 nm, according to the maximum peak observed in the CD spectrum analysis.

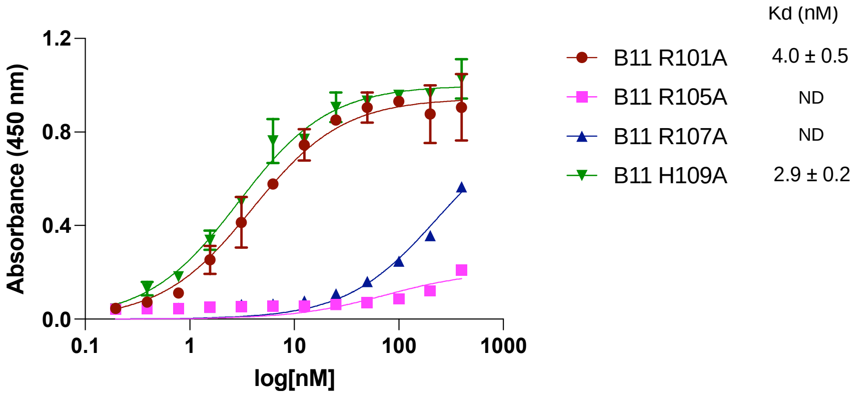

Figure S3. ELISA binding curves of four different SG4 mutants to MycG4: R101A (red), R105A (pink), R107A (blue), and H109A (green). Dissociation constants (Kd) are indicated in nanomolar, in some cases they could not be determined (ND). Error bars represent the Standard Error of the Mean (s.e.m.) calculated from two replicates.

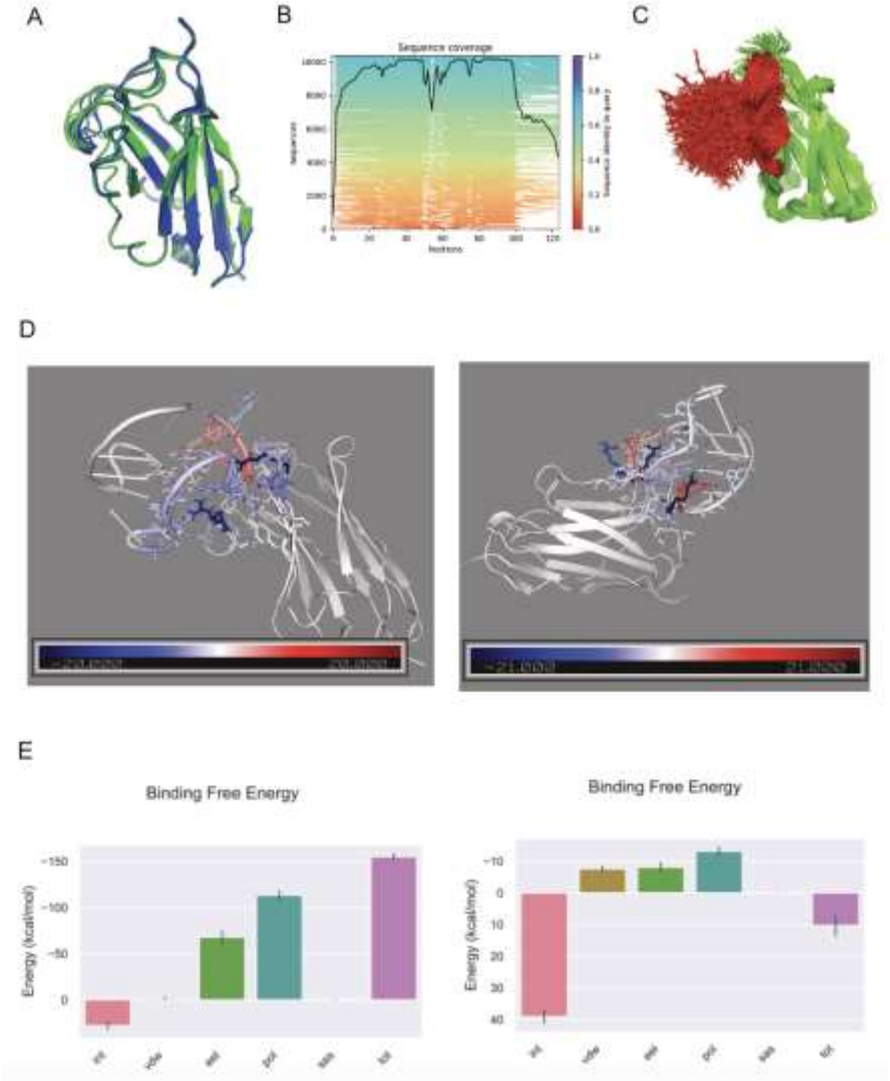

Figure S4. (A) Overlap of all relaxed predicted structures of SG4 (blue) and mSG4-R105A (green). (B) Alphafold2 similarity score and sequence coverage (PAE) of SG4 nanobody produced during the Alphafold2 prediction. (C) Overlap of SG4 nanobody structures every 10 ns of molecular dynamics for 300 ns, CDR3 in red sticks demonstrating the flexibility of the region. (D) Represented complexes of the two clusters resulted from docking experiments and used in further molecular dynamics. (E) R105A (left) and H109A (right), binding energy delta and per component decomposition of energy contribution at residues 105 and 109.

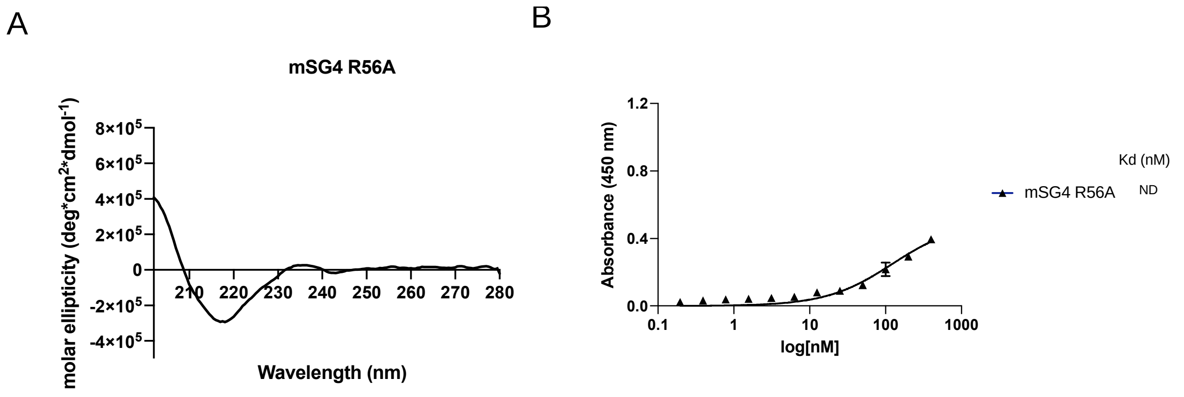

Figure S5. (A) mSG4 R56A secondary structures determined by CD spectroscopy (200-280 nm) showing a characteristic β-sheet with a negative peak at 218 nm and a positive peak at 200 mm. Units are measured in molar ellipticity. (B) ELISA binding curve of SG4 mutant R56A to MycG4. The dissociation constant (K_d) could not be determined (ND). Error bars represent the Standard Error of the Mean (s.e.m.) calculated from two replicates.

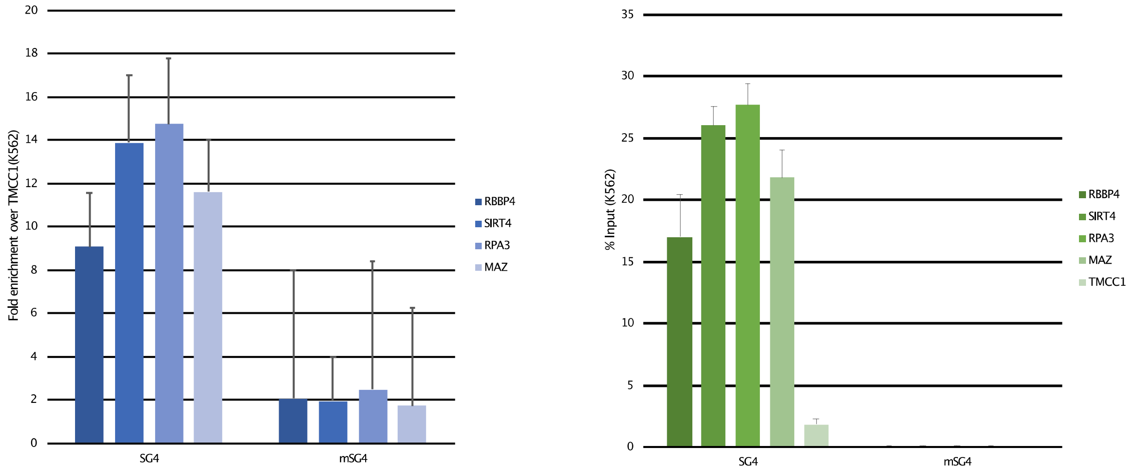

Figure S6. Bar plot of the enrichment of the amplification through qPCR and bar plot of the percentage of the DNA input recovered (% Input) by either SG4 ChIP or mSG4 R105A in the same genomic regions, established by qPCR, of four different G4-positive controls (RBBP4, SIRT4, RPA3, MAZ) over a G4-negative region (TMCC1), indicated by individual bars. The group of left bars is relative to SG4 ChIP-seq, while the right one mSG4 R105A ChIP in K562.

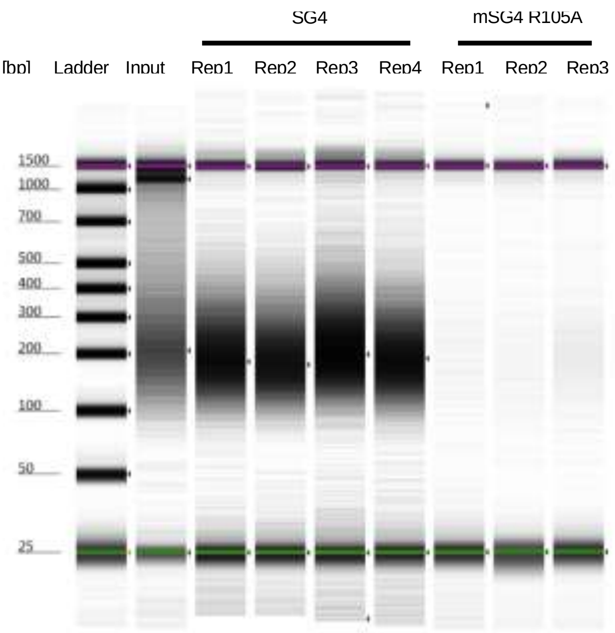

Figure S7. Trace of the fragment sizes in bp of DNA fragments pulled down by either SG4 or mSG4 R105A ChIP (Tapestation).

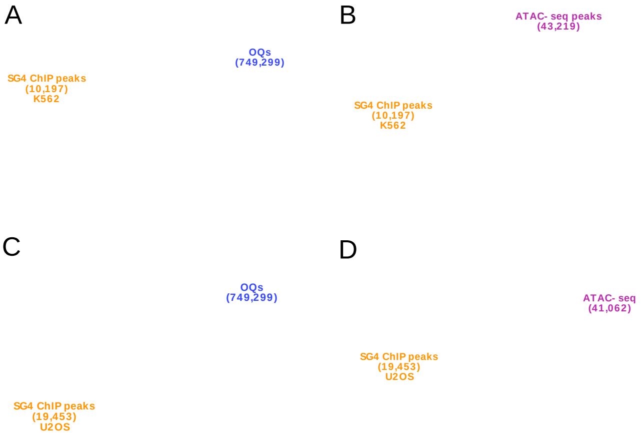

Figure S8. Venn diagrams of the overlap of SG4 regions identified through ChIP-seq (SG4 ChIP-seq peaks) with sequences previously identified as capable of folding into a G4 structure *in vitro* (so-called observed G4 sequences and referred to as OQs) (4) in K562 (A) and in U2OS (C). Venn diagrams of the overlap of SG4 ChIP-seq peaks with accessible chromatin sites identified through Assay for Transposase Accessible chromatin with high-throughput sequencing (ATAC-seq peaks) in K562 (B) and in U2OS (D) (1). Total numbers of the total regions identified by SG4, OQs, and ATAC-seq peaks, and number and percentage of SG4 peaks overlapping with OQs or ATAC-seq peaks are reported for both cell lines.

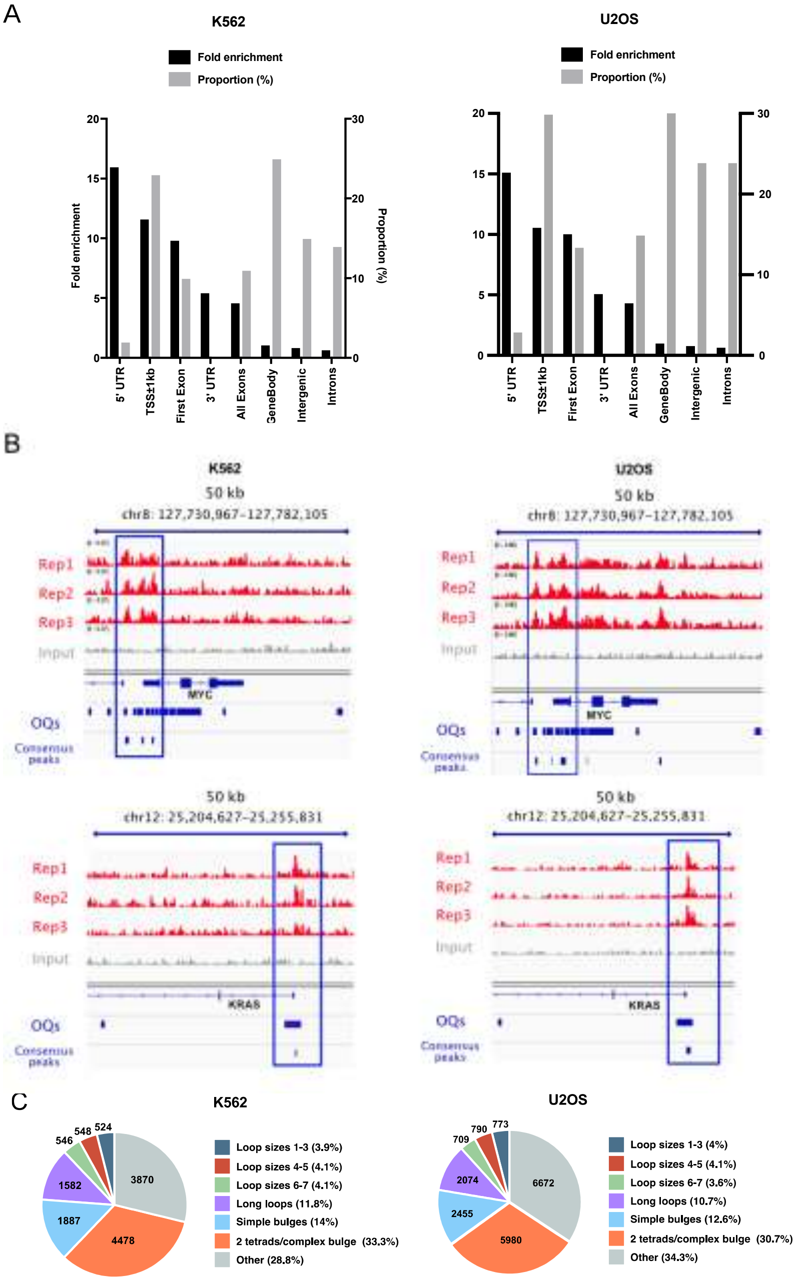

Figure S9. (A) Fold enrichment over random (black bars) and proportion (grey) of SG4 ChIP-seq consensus regions across different genomic features in K562 and U2OS cell lines. (B) Genome browser screenshots of one of the three technical replicates per biological replicate of SG4 ChIP-seq (red) and input (grey) tracks at the promoters of *MYC* and *KRAS* genes in K562 and U2OS. OQs and consensus regions across two of the three SG4 ChIP-seq replicates are shown. (C) Total number of regions showing G4 structural motifs, percentage in brackets of G4 structural motifs detected by SG4 in K562 and U2OS. Loop sizes 1-3 (dark blue), 4-5 (red), 6-7 (green) are G4s that have one or more loops of this length in their motif; a G4 with at least a loop longer than 7 bases is referred as a long loop (purple); a G4 is called a simple bulge (light blue) when it contains a bulge of 1-7 base; 2-tetrads/complex bulge (orange) are G4s composed of only two tetrads or with many 1-5 base bulges; the definition of "other" (grey) refers the sequences could not be described by using the categories previously mentioned.

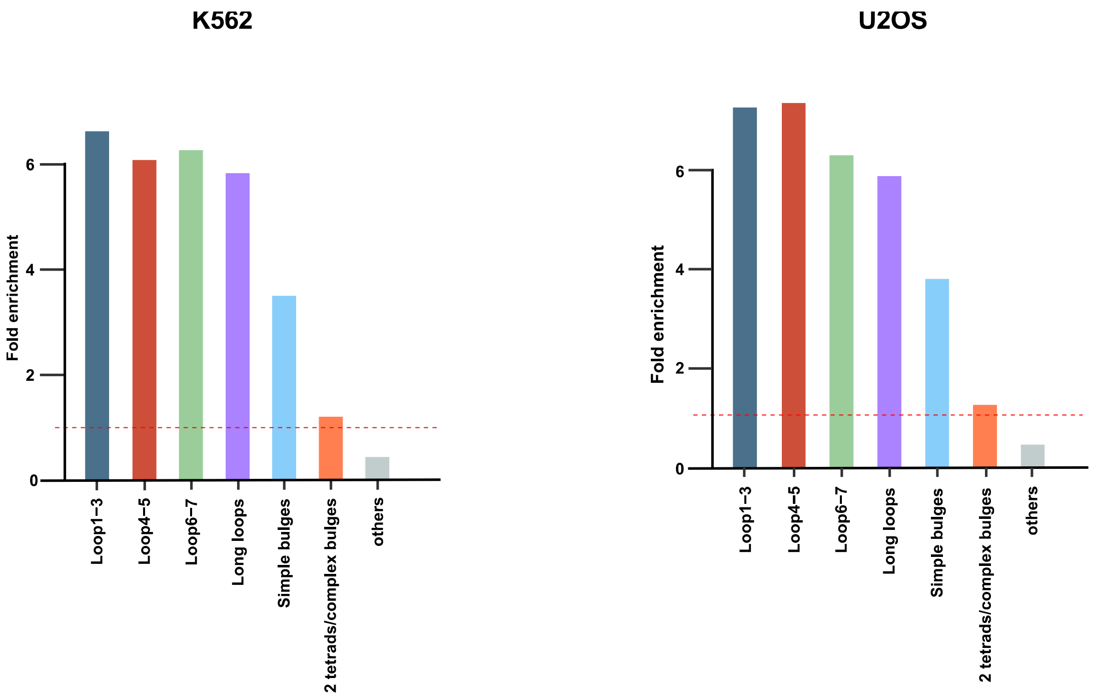

Figure S10. Fold enrichment (obtained as actual observed occurrences over average of 10 randomizations) of G4 structural motifs as defined in (4) described in Figure 3 detected by SG4 ChIP-seq in K562 and U2OS. Enrichment values above 1 (red dotted line) indicate enrichment of the motif over random expectation.

Table 1. The biotinylated oligonucleotide sequences.

Oligonucleotide name	Sequence
MycG4	5' – TGA GGG TGG GTA GGG TGG GTA A – 3'
Kit1G4	5' – AGG GAG GGC GCT GGG AGG AGG G – 3'
VegfG4	5' – CGG GGC GGG CCT TGG GCG GGG T – 3'
hTeloG4	5' – GGT TAG GGT TAG GGT TAG GGT TAG GGT TAG – 3'
TbaG4	5' – GGT TGG TGT GGT TGG – 3'
Mutant Myc	5' – TGA GTG TGC GTA GTG TGT GTA A – 3'
dsDNA	*sense* 5'- TGAGTGTGTGTAGTGTGTGTAA – 3'

REFERENCES

1. Shen J, Varshney D, Simeone A, Zhang X, Adhikari S, Tannahill D, et al. Promoter G-quadruplex folding precedes transcription and is controlled by chromatin. Genome Biol. 2021;22(143):1–14.
2. Hänsel-Hertsch R, Spiegel J, Marsico G, Tannahill D, Balasubramanian S. Genome-wide mapping of endogenous G-quadruplex DNA structures by chromatin immunoprecipitation and high-throughput sequencing. Nat Protoc. 2018;13(3):551–64.
3. Kirschner K, Samarajiwa SA, Cairns JM, Menon S, Pérez-Mancera PA, Tomimatsu K, et al. Phenotype Specific Analyses Reveal Distinct Regulatory Mechanism for Chronically Activated p53. PLoS Genet. 2015;11(3):1–28.
4. Chambers VS, Marsico G, Boutell JM, Di Antonio M, Smith GP, Balasubramanian S. High-throughput sequencing of DNA G-quadruplex structures in the human genome. Nat Biotechnol. 2015;33(8):877–81.
5. Hui WWI, Simeone A, Zyner KG, Tannahill D, Balasubramanian S. Single-cell mapping of DNA G-quadruplex structures in human cancer cells. Sci Rep. 2021;11(23641):1–7.
6. Meers MP, Tenenbaum D, Henikoff S. Peak calling by Sparse Enrichment Analysis for CUT&RUN chromatin profiling. Epigenetics and Chromatin. 2019;12(42):1–11.
7. Mirdita M, Schütze K, Moriwaki Y, Heo L, Ovchinnikov S, Steinegger M. ColabFold: making protein folding accessible to all. Nat Methods. 2022;19:679–82.
8. Páll S, Zhmurov A, Bauer P, Abraham M, Lundborg M, Gray A, et al. Heterogeneous parallelization and acceleration of molecular dynamics simulations in GROMACS. J Chem Phys. 2020;153(134110):1–15.
9. Scherer MK, Trendelkamp-Schroer B, Paul F, Pérez-Hernández G, Hoffmann M, Plattner N, et al. PyEMMA 2: A Software Package for Estimation, Validation, and Analysis of Markov Models. J Chem Theory Comput. 2015;11(11):5525–42.
10. Van Zundert GCP, Rodrigues JPGLM, Trellet M, Schmitz C, Kastritis PL, Karaca E, et al. The HADDOCK2.2 Web Server: User-Friendly Integrative Modeling of Biomolecular Complexes. J Mol Biol. 2016;428(4):720–5.
11. Honorato R V., Koukos PI, Jiménez-García B, Tsaregorodtsev A, Verlato M, Giachetti A, et al. Structural Biology in the Clouds: The WeNMR-EOSC Ecosystem. Front Mol Biosci. 2021;8(729513):1–7.
12. Valdés-Tresanco MS, Valdés-Tresanco ME, Valiente PA, Moreno E. Gmx_MMPBSA: A New Tool to Perform End-State Free Energy Calculations with GROMACS. J Chem Theory Comput. 2021;17(10):6281–91.
